# Computer work and musculoskeletal disorders of the neck and upper extremity: A systematic review

**DOI:** 10.1186/1471-2474-11-79

**Published:** 2010-04-29

**Authors:** Morten Wærsted, Therese N Hanvold, Kaj Bo Veiersted

**Affiliations:** 1 National Institute of Occupational Health, PO Box 8149 Dep, N-0033 Oslo, Norway

## Abstract

**Background:**

This review examines the evidence for an association between computer work and neck and upper extremity disorders (except carpal tunnel syndrome).

**Methods:**

A systematic critical review of studies of computer work and musculoskeletal disorders verified by a physical examination was performed.

**Results:**

A total of 22 studies (26 articles) fulfilled the inclusion criteria. Results show limited evidence for a causal relationship between computer work per se, computer mouse and keyboard time related to a diagnosis of wrist tendonitis, and for an association between computer mouse time and forearm disorders. Limited evidence was also found for a causal relationship between computer work per se and computer mouse time related to tension neck syndrome, but the evidence for keyboard time was insufficient. Insufficient evidence was found for an association between other musculoskeletal diagnoses of the neck and upper extremities, including shoulder tendonitis and epicondylitis, and any aspect of computer work.

**Conclusions:**

There is limited epidemiological evidence for an association between aspects of computer work and some of the clinical diagnoses studied. None of the evidence was considered as moderate or strong and there is a need for more and better documentation.

## Background

Musculoskeletal complaints in the neck and upper extremity and computer work are common in modern society and both show an increasing trend. Several previous reviews have indicated a possible causal relationship between computer work and musculoskeletal complaints in the neck and arm [1-9]. The epidemiological studies concerning computer use and musculoskeletal health are mainly based on subjective measures of upper extremity musculoskeletal symptoms. This may give important knowledge with regard to preventing these ailments. However, when evaluating a possible causal relationship between computer work and musculoskeletal disorders, such as when handling insurance claims, it is necessary with a more objective measure of a sustained effect on the musculoskeletal system and this is the basis for the present review. In Norway this topic has a special interest at present (2010), as the government investigates the possibility to include specific musculoskeletal disorders on the list of occupational diseases that may receive compensation.

Physical factors, psychosocial and organizational factors as well as individual factors are all thought to affect the workers musculoskeletal health [10]. A complex of various environmental work factors characterizes computer work, but we evaluate all psychosocial and organizational factors as well as individual factors to be common for all kinds of working environment and not specific for computer work [5]. This review will therefore mainly focus on the specific physical factors relevant to computer work when evaluating a possible causal relationship to neck and upper extremity musculoskeletal disorders.

Computer work is here defined as work with video display units (VDU) or video display terminals (VDT) that involves the use of keyboard and/or mouse. Work that involves the use of a personal digital assistant, handheld computer, personal organizer device or similar forms of small size mobile computers is not considered in this review. Carpal tunnel syndrome (CTS) in relation to computer work is the specific topic of a parallel review [11] and is not included in the present review. These two reviews are mainly based on scientific reports [12,13] made on behalf of the Scientific Committee of the Danish Society for Occupational and Environmental Medicine for the use of the Danish National Board of Industrial Injuries http://www.ask.dk in their evaluation of whether specific musculoskeletal disorders in computer workers should be included on their list of occupational injuries and diseases that may be compensated through the Danish Worker Compensation Act. The published review on CTS [11] is updated till August 2008 and the present review is updated till February 2010.

The aim of the present study is to critically review the epidemiological evidence for a possible causal relationship between different aspects of computer work, including keyboard and mouse use, and neck and upper extremity musculoskeletal disorders diagnosed with a physical examination.

## Methods

### Literature search

A computer based literature search was performed in February 2010 in the five internet databases PubMed, EMBASE(Ovid), ISI Web of Science, CSA Health and Safety Science Abstracts, and OSH References Collection Search (giving access to the following six databases OSHLINE, NIOSHTIC, NIOSHTIC-2, HSELINE, CISILO and Canadiana). The search string consisted of three parts to cover musculoskeletal disorders, body region and computer work. At least one key term from each of these three parts had to match. The first part on musculoskeletal disorders had general terms, such as disorder, diagnosis, pain, etc, more specific terms such as tendonitis, tendinitis, tendonopathy, etc, and specific disease entities such as epicondylitis, tennis elbow, tension neck syndrome, etc. Altogether this part covered 40 distinct terms (the actual number of key terms in the search in each database varied and could be greater, as a consequence of the structure and logic of the search engine). The second part defined the neck and upper limb with eleven distinct terms, and the last part defined computer work with eighteen terms. To maximize the number of relevant studies retrieved, a search was also done in the authors personal files in addition to screening the reference lists of all included studies and six selected reviews [1,3,5-8].

The following inclusion criteria were applied: (i) the study should be peer reviewed and published in English (reports, abstracts and proceedings were not included), (ii) the study gave data on computer use in a working age population, (iii) the study had to include a relevant objective examination (e.g. a physical examination, scanning, or x-ray) of musculoskeletal disorders in the neck and upper extremity (except carpal tunnel syndrome), and (iv) the publication from the study had to relate the exposure to computer work to the findings of the objective examination. Results on the effect of treatment or other interventions on musculoskeletal disorders in computer users were not included.

### Quality assessment

The included articles were assessed with respect to their methodological strength, based on existing quality criteria from a previous review of work-related risk factor for neck pain [14]. The present quality assessment list included the same items as used by Ariëns and co-workers [14], but had an extra item added (item 23 [see Additional file 1]). Each of the items were scored either positive (1), negative (0), or not applicable. The number of items that were applied differed slightly between articles depending on the study design [see Additional file 1]. Two of the authors (KBV and MW) independently scored each of the articles. The final assessment [see Additional file 1] was decided in a consensus meeting with all three authors. The conclusion on methodological quality was divided into low (below 50% positive scores), moderate (50-65), high (65-80), or very high (above 80%) quality. It can be argued that the use of scores from a quality assessment list may be misleading by lacking a possibility for a more thorough evaluation of the way the objective of the different items has been met and a lack of an importance weighting of the single items. The authors therefore considered each paper with respect to the result of schematic quality rating, by doing an overall non-systematic evaluating of the studies face validity, as well as their strengths of methodology and analyses. However, this evaluation did not bring forth obvious misclassifications of papers into low, moderate or high methodological quality and thus the more transparent schematic quality rating is used in the evaluation.

### Level of evidence

To evaluate the evidence of causality in this review, the criteria from the NIOSH review in 1997 [15] is used as the basis. These are in turn built upon the criteria of causality suggested by Hill [16]. We used the following four evaluation criteria:

1. Consistency: an association that is repeated in multiple independent studies supports the plausibility of a causal relationship. The causal relationship is weakened when comparable studies show different results.

2. Temporality: exposure always precedes the response in time. This is ensured in prospective cohort designs.

3. Exposure-effect relationship: an association between the occurrence of a disease and the intensity, duration or frequency of the exposure, will support a causal relationship.

4. Coherence of evidence: a consistence of the associations and the natural history and biology/physiology of the disorder (biological plausibility) will support a causal relationship.

Findings that met all of the causality criteria were emphasised more than findings that met few of the criteria. On the basis of this assessment the degree of evidence was decided.

On the basis of IARC's classification system the strength of evidence from the selected epidemiological articles was classified into 5 categories, according to the categories suggested by The Scientific Committee of the Danish Society of Occupational and Environmental Medicine, 2005 (referred in Jensen 2008 [17]). The following categories were used:

+++ Sufficient evidence of a causal association

++ Moderate evidence (bias and confounding are not a likely explanation of associations (<50%))

+ Limited evidence (bias and confounding are not an unlikely explanation of associations (>50%))

0 Insufficient evidence of a causal association

- Evidence suggesting lack of a causal association

The level of evidence was reduced by at least a step if only one study showed a significant effect, even if the study was of moderate/high quality.

### Definition of neck and upper extremity disorders with physical findings

The relevant disorders are selected on the basis of findings in the epidemiological studies included in this review. The demands on precision of the diagnostic tests are lower for the epidemiological studies compared to what is needed in most clinical practice [18,19]. In an additional file [Additional file 2] the definitions of the diagnoses used by the included studies are presented.

## Results

After exclusion of duplicates, papers in non-English languages and papers that covered non-relevant topics from the ca 26,000 titles retrieved through the computerized data base search, approximately a thousand abstracts were evaluated for relevance and nearly two hundred epidemiological papers were read in full. A total of 22 studies published in 26 papers fulfilled the criteria for inclusion [20-45]. Eleven studies were prospective, either in a traditional follow-up design [24,25,29,31,32,37-39,42] or the effect of an intervention in the work place was followed [20,21,26,27,36,40], and two studies had a case-control design [23,43], whereas nine studies had a cross-sectional design [22,28,30,33-35,41,44,45]. However, in most of the prospective studies, cross-sectional data analyses are also presented. Even if the examination protocols may specify a number of musculoskeletal diagnoses, the results are to a great extent presented with several diagnoses grouped together by anatomical region (i.e. neck/shoulder disorders) due to few cases in each diagnostic category. In six of the included studies [20,27,30,34,36,41] clinical signs such as restricted movement or pain provoked by the examination were mostly reported instead of specific diagnoses. All studies included in this review were retrieved through the computerized search, except one [43] that was retrieved from personal files.

Three tables provided as additional files give the details of the schematic quality assessments [Additional file 1], give an overview of the case definitions and clinical examinations used in the included studies [Additional file 2], and summarize their results [Additional file 3]. In these three tables the studies are listed alphabetically according to the first author (or the acronym NUDATA for the three papers in that study). The same order is followed when the studies are shortly commented below.

In a multifaceted ergonomic intervention study on three groups of 30 data workers each, Aarås and co-workers [20] revealed no changes in clinical signs during the one year follow-up period. At baseline, however, there were differences in some clinical signs between the three groups of workers, with more signs among the female workers doing monotonous data entry work, compared to both female and male workers with more varied data dialogue work [Additional file 3]. The paper had a high methodological quality, with a positive score on 67% of relevant items in the schematic quality rating [Additional file 1].

Arvidsson and co-workers [21] studied air-traffic controllers before and after a change (in March 2005) from varied computer work to a strictly mouse-based system. The study had a very high quality with positive score on 83% of relevant items in the schematic quality assessment [Additional file 1]. With the new system the controllers had lower variation of work postures and less rest in the forearm extensor muscles, as assessed by technical measurements [46]. Musculoskeletal diagnoses in the elbow-hand region increased significantly. If the analysis was limited to the younger half of the air-traffic controllers, musculoskeletal disorders in the shoulder, neck and upper back also showed an increase [Additional file 3]. The predominance of right arm disorders was more pronounced at follow-up (analyzed with shoulder, elbow and hand diagnoses grouped together).

In a recent study comparing a 100 bank workers with extensive computer use with 65 office workers with less than 2 h/day of computer use, Aydeniz and Gursoy [22] found that the extensive computer users had more positive clinical tests for diagnoses in the shoulder-neck, as well as in the elbow and wrist [Additional file 3]. However, this cross-sectional study was not very well documented and rated positive on less than half (44%) of the items of the quality check-list [Additional file 1] and we concluded with a low methodological quality.

Baker and co-workers [23] studied personal keyboard use patterns in subjects with and without musculoskeletal disorders of the upper extremity, utilizing an advanced video-based observation instrument [47,48]. Their subjects were recruited from a university faculty and staff, and the study is in the setting of this review a case-control study. However, as this study was designed to evaluate the observation instrument's ability to discriminate between computer workers with and without musculoskeletal disorders, and not designed as an ordinary epidemiological case-control study, the study obtained a relatively low score on methodological quality (50% items positive [Additional file 1]). Subjects with upper extremity musculoskeletal disorders worked with a greater neck flexion angle [Additional file 3]. However, the study design with only cross-sectional exposure data did not allow a conclusion on the causal direction of this association.

In a prospective study of a cohort of office workers with data collection in 1981 and 1987 Bergqvist and co-workers performed a comprehensive evaluation of the physical work environment and the subjects' musculoskeletal problems. As an outcome measure at the last data collection in 1987 a thorough physical examination by a physiotherapist of current computer users was included in addition to questionnaire based subjective complaints, defining several musculoskeletal disorders of the neck, shoulder and arm. Two papers [24,25] present the data relevant for this review. The study was concluded as having a high quality (72% of items positive [Additional file 1]). Most of the analyses with musculoskeletal disorders as health outcome were cross-sectional, with the exposure data also collected in 1987, but a measure of total accumulated hours of VDU work was calculated based on the data from both surveys. There were no simple relationships between the amount of computer work per se and musculoskeletal disorders in the neck, shoulder or arm/hand. However, when an additional workplace factor such as use of forearm support was included in the analyses, some relationships were found [Additional file 3].

Conlon and co-workers [26] performed a randomised controlled intervention trial with alternative computer mouse and forearm support board in a group of 206 engineers from a large aerospace engineering firm. The cohort consisted of approximately 70% males and was followed for a year. The subjects were randomized into four groups, receiving either one or both interventions or continuing with the conventional computer workstation. The subjects were assessed each week for musculoskeletal discomfort and those reporting a certain level of discomfort were referred to a physical examination with a protocol assessing for the presence of 40 upper extremity and neck musculoskeletal disorders. The study had a very high methodological quality and rated positive on 89% of the checklist items [Additional file 1]. Forty-two incident cases of musculoskeletal disorders were identified during the follow-up period, however, they found no associations with either one of the interventions to the incidence of these shoulder-neck or arm-hand disorders [Additional file 3].

Dainoff and co-workers [27] performed an intervention trial in a group of 28 female data entry operators, including an advanced workstation redesign, ergonomic training and optometric corrections. The subjects were assessed with a physical examination one month and one year post-intervention. The examination consisted among others of measurements of the range of passive movements, tenderness or pain upon provocation (movements, palpation or endurance test), and palpation of trigger points in the trapezius. The study was assessed as having a moderate quality with 56% positive items in the schematic assessment [Additional file 1]. The authors found a decrease of positive signs and trigger points in the shoulder at the one month test following the intervention, and this change was still present at one year follow-up [Additional file 3].

Ferraz and co-workers [28] compared keyboard operators with traditional office workers. Exposure data were obtained from self report and from registered keystroke performance in the previous month. The keyboard operators did not use a computer mouse (evaluated from photos in the paper showing subjects at the computer workstations). All subjects received an examination by a physiotherapist, and subjects reporting symptoms on self report or positive signs on the examination, received a full examination by a rheumatologist. The study concluded that keyboard use was associated with tension neck syndrome, shoulder tendinitis and wrist tendinitis [Additional file 3]. However, the study had several methodological shortcomings and scored only positive on 50% of the quality assessment items [Additional file 1] and was concluded as having a moderate quality.

Ferreira and co-workers [29] identified retrospectively for a two and a half year period the monthly incidence of musculoskeletal disorders in a dynamic cohort of call centre operators in a banking subsidiary. From medical records and reconstruction of changes in administrative and technical procedures influencing the operators workload, the authors concluded that the incidence of musculoskeletal disorders in the wrist and hand was reduced both when a work schedule with 10 minutes break per hour computer work was introduced, and when the goal on average time to answer a call was reduced [Additional file 3]. However, as the previous study, this study had methodological shortcomings and a moderate quality with only 50% positive items on the quality check list [Additional file 1].

A cross-sectional study by Fogg and Henderson [30] compared 512 computer users with 561 clerical workers not using a computer. The study had a low quality with only 19% positive items in the schematic quality rating [Additional file 1]. They concluded that repetitive strain was more frequent among computer users, who also had their pain condition for a longer period [Additional file 3].

Gerr and Marcus and co-workers [31,32] measured 20 different characteristics of the workstation lay-out and work postures adopted of 632 newly hired computer workers. The workers were followed for up to three years. This very high quality comprehensive prospective study had 94% positive items in the schematic quality assessment [Additional file 1]. The subjects filled in a diary each work day documenting computer use and incident musculoskeletal symptoms. All subjects reporting symptoms were examined for specific musculoskeletal disorders. Hours of computer work per week were associated with hand-arm disorders, but not with shoulder-neck disorders. Some of the ergonomic workstation characteristics showed an association with either shoulder-neck or hand-arm diagnoses [Additional file 3], such as a protective effect on hand-arm disorders of having at least some free space between the keyboard and the table edge, and a negative effect of a wrist rest. However, the majority of the recorded ergonomic exposure variables did not show any significant association to musculoskeletal disorders. The hazard ratios shown in [Additional file 3] are from the final model. The paper also presents unadjusted and covariate adjusted hazard ratios for several other of the postural risk factors, i.e. showing that the presence of a chair armrest tended to have a protective effect against neck-shoulder disorders (covariate adjusted HR = 0.65 (0.39-1.08)). Somatic pain syndrome with a definition similar to the more common diagnosis tension neck syndrome [Additional file 2] constituted 87% of all diagnoses in the shoulder-neck and was found in 95% of the subjects with one or more diagnoses in the shoulder-neck region.

Hales and co-workers [33] performed a comprehensive cross-sectional study on 533 telecommunication workers using a computer for at least 6 hours per workday. The study had a high quality with a positive score on 69% of the checklist items [Additional file 1]. All subjects, regardless of symptom status, were offered a physical examination by a physician. There was no association between estimated keystrokes per day and musculoskeletal disorders.

Hünting and co-workers [34] in an old cross-sectional study compared computer workers, full time typist and traditional office workers who rarely used a keyboard. The study had several methodological shortcomings and with present day standards a low quality with only 19% positive scores on the checklist items [Additional file 1]. They found more pressure pain and painfully limited head movements in data entry work, compared to traditional office work and that an increased ulnar deviation in keyboard use was associated with clinical findings [Additional file 3].

Jepsen and Thomsen [35] studies the relation between subjective symptoms and clinical findings of peripheral neuropathy of the upper limbs of a sample of computer users. They present their data separate for mouse operating and non-mouse operating limbs. This within subject comparison makes the study relevant for our review. However, the scope and aim of this study is far from the typical epidemiological study in this review, resulting in lower quality score than "deserved" with only 44% positive checklist items [Additional file 1]. For all three predefined characteristic patterns of physical findings, more mouse operating than non-mouse operating limbs fulfilled the criteria [Additional file 3]. The paper does not discuss to what extent this difference is attributable to mouse use and not to a general difference between dominant and non-dominant limbs.

Konarska and co-workers [36] studied an intervention on a group of 33 data entry workers, using an examination protocol shared with studies by Aarås and co-workers [20] and Dainoff and co-workers [27] (both referred above), the studies in the collaboration being compared in a separate paper [49]. However, the Konarska study had more practical and technical difficulties and a high dropout rate. These facts, among others, contributed to a low quality with only 28% positive items on the checklist [Additional file 1] and, possibly, to the fact that they found no changes in clinical findings from before till after the intervention.

The NUDATA study (acronym for Neck and Upper extremity Disorders Among Technical Assistants) recruited a cohort of 6943 technical assistants and machine technicians from the Danish Association of Professional Technicians, representing a population with a wide distribution of both mouse device usage and keyboard usage. The cohort was followed for a year with a fairly high response rate at follow-up [Additional file 3]. The data relevant for this review was presented separately in three papers according to anatomical region: neck and shoulder disorders by Brandt et al. [37], forearm disorders by Kryger et al. [38] and elbow and wrist/hand disorders by Lassen et al. [39]. The study had a high quality (rated 78% of items positive [Additional file 1]) and had self-reported exposure data for both computer mouse use and keyboard use, and for several ergonomic factors [Additional file 3]. The authors have in a separate paper [50] validated the data on self-reported durations for computer activities and shown them to be quite inaccurate. Subjects who reported subjective symptoms of musculoskeletal problems in the neck, shoulder or arm were invited to a clinical examination. In the baseline survey the odds of being a clinical forearm case were increased for participants using mouse > 30 h/week [38]. They also identified a possible dose-response association between hourly mouse use and tension neck syndrome, however, the corresponding analysis for hourly keyboard use only gave a very weak and not significant association [37]. In a baseline contingency table analysis only published in a PhD-thesis based on the NUDATA-study [51], a significant association was found between mouse time and wrist tendonitis (extensor side, test for trend: p = 0.02) and near significant association between mouse time and the clinical forearm case diagnosis (test for trend: p = 0.08). They did not find other statistical significant associations between the clinical conditions studied and the ergonomic factors or weekly hours of keyboard or mouse use [Additional file 3]. For tension neck syndrome the use of arm support tended to be protective with regard to mouse use, but had no effect with regard to keyboard use, and a variable labelled 'abnormal mouse position' seemed to be protective [37]. At baseline there were too few cases of several of the clinical entities to make a reliable analysis possible, and in their one year follow-up, the number of incident clinical cases was too low in all diagnostic categories. An important strength of this study was the very big study base and the fairly good response rates, giving information on prevalence and one-year incidence of common musculoskeletal disorders among computer workers. A drawback, however, that this study shares with several of the other studies in this review, is the fact that only subjects reporting subjective symptoms were invited to the clinical examination, leaving us with no knowledge of the possible occurrence of the studied clinical conditions in the subjects not filling the criteria for being a symptom case.

Rempel and co-workers [40] followed a one year randomized controlled intervention trial with a trackball alternative mouse and/or a forearm support board among call centre operators. They used the same examination protocol as Conlon and co-workers [26] (referred above), and had the same very high study quality (89% positive checklist items [Additional file 1]). They found that the forearm support board intervention was associated with a reduced incidence of shoulder-neck diagnoses and a tendency for a similar effect on hand-arm diagnoses, and that the trackball intervention was associated with a reduced incidence of arm-hand diagnoses [Additional file 3].

Motivated by the Australian "epidemic" of computer related musculoskeletal disorders in the early 1980's, Ryan and Bampton [41] studied thoroughly a group of data process operators. However, the reporting of the study has several shortcomings, i.e. the number of males and females among the 143 subjects was not stated, and the study obtained a low quality score with 38% of items positive [Additional file 1]. The main analysis in the paper is a comparison of the 41 subjects with the highest upper limb symptoms score and the 28 subjects with the lowest score. The score is based on symptoms and signs from the neck and upper limbs. Some significant differences between these two subgroups were found, among them three measures of the subjects working posture at the computer workstation [Additional file 3].

A group of call centre operators was followed by Toomingas and co-workers [42] with monthly questionnaires for nearly a year and was compared to a large reference group of computer users from other professions. Subjects reporting symptoms from the musculoskeletal symptoms, but were free of symptoms the preceding month, were invited to a medical examination. However, more than half the female call centre operators had symptoms at baseline and all follow-ups and thus never qualified as an incident symptom case. Among the incident cases receiving an examination, the call centre operators had a higher incidence of wrist/hand diagnoses and of conditions with nerve tissue involvement [Additional file 3]. Unfortunately, the study is not very well documented, and is assessed to have a moderate quality with 50% of items positive [Additional file 1].

Tornqvist and co-workers [43] performed a community based case-control study to assess the influence of work-related factors on seeking care for neck or shoulder disorders. The study had a high methodological quality, with 70% of the quality items positive [Additional file 1]. From 1994 to 1997 they sampled 392 cases (274 females) and 1511 controls. A question on computer work 4 hours or more per work day was included in the questionnaire on work-related exposure factors, and this factor was associated with an increased risk for shoulder-neck diagnoses in women [Additional file 3].

Turhan and co-workers [44] have recently published this cross-sectional study of computer workers. The study is comprehensive, but the presentation, data-analysis and discussion are not of a very high standard, being reflected in a low quality with only 44% checklist items positive [Additional file 1]. In their univariate analysis they showed significant associations between observed awkward working postures and diagnosed musculoskeletal disorders [Additional file 3].

In a community based study with data collection in 1998-2000 Walker-Bone and co-workers [45] studied specific upper limb disorders and non-specific pain states. Questionnaires were mailed to the working age population of two general practices, identifying subjects with pain in the neck or upper extremity who were offered a standardized physical examination. Of interest were subjects with persistent shoulder, elbow or wrist pain, which should be due either to a specific musculoskeletal disorder (n= 250) or to non-specific pain (n = 176), excluding 70 subjects with a mixed pattern. The subjects reporting no neck or shoulder pain in the questionnaire served as a reference population (n = 2248). The study had a moderate methodological quality, with 63% positive items on the quality checklist [Additional file 1]. The postal questionnaire assessed exposure factors and had a question on daily keyboard or typewriter use, finding that using keyboard or typewriter more than one hour pr day was associated with an increased risk of wrist tenosynovitis [Additional file 3].

## Discussion

In the present review on musculoskeletal disorders in computer work, a main inclusion criterion was that the disorders had to be documented by some sort of physical examination, and not solely based on subjective reports. This limits the number of studies available, and we were only able to find 26 peer reviewed papers fulfilling all inclusion criteria. The computerized data base search may have its limitations, as the large number of irrelevant titles obtained may illustrate. Only one of the included studies was not found in the data base search. This may indicate that the search probably has found the important studies in the field. This is supported by the fact that checking the reference lists of included papers and selected reviews did not bring forth any extra studies. However, it may not be ruled out that smaller studies or studies that have recently been published in journals not commonly used by researchers in this field have been missed. The one study was missed in the search because computer use as a risk factor was not among the most important occupational risk factors reported and thus not mentioned in keywords, title or abstract. This may be the case also for other community based studies. The fact that only studies published in English are included is a limitation. There are good studies published in scientific journals written in e.g. German, French and Japanese. However, our impression is that at least in the last 10 to 20 years, the largest and most important studies have one or more publications in English language journals. A risk of a publication bias exists, but it is not obvious. If present, we would expect studies with moderate to low quality not showing an association between exposure and outcome to a lesser extent were published compared to studies showing an association.

The quality of the 22 included studies varies a lot, as the schematic scoring may illustrate [Additional file 1]. The relevance of the studies may also vary. Some of them are fairly old, with data from computer workstations and computer work that probably is not common today. The ergonomics of the computer workstations and the computer work-tasks themselves may also vary a lot at present between different parts of the world and between different occupations. However, the intensive interaction between man and computer, that is the hallmark of modern office work, is shared by all studies included. For some of the included studies the aim is rather different from the aim of our review and may thus have a study design, data presentation and/or data analysis that is not optimal for our purpose. However, as long as a paper fulfils the inclusion criteria and gives results that may shed light on our research question, the paper is included and evaluated in our context.

In all included studies the physical examination was a clinical examination performed by a physician, a physiotherapist or another trained health professional, even if other objective examinations such as scanning or x-ray also would satisfy the inclusion criteria. However, the examination protocols and the concluding diagnoses or signs differed substantially between the included studies [Additional file 2]. The same is true with regard to the characterization of the exposure to computer work and the way the study populations were selected. Even to the extent that the different studies have used the same or similar definitions for exposure and effect, the prevalence and incidence of specific musculoskeletal disorders have in most studies been so low that the diagnoses have been grouped together in order to have enough cases in each category in the statistical analysis. This is a challenge when attempting to summarize and draw conclusions on the relationship between computer work and musculoskeletal disorders of the neck and upper extremity. Ideally we should be able to weigh evidence for a possible relationship between specific disorders and (aspects of) computer work, as e.g. a nerve compression condition and a bursitis or tendonitis may be caused or aggravated by different causal mechanisms. We will however to a great extent be limited to evaluate musculoskeletal disorders of a given body region. An additional problem is a lack of consensus on definitions for musculoskeletal disorders and on the clinical examination necessary to conclude, which as mentioned also can be seen in the papers included in this review. When the prevalence or incidence of a common musculoskeletal disorder show a very big variation, as there also are examples of in the present review, one may suspect that this possibly could be an artifact due to different clinical criteria, making it even more difficult to compare prevalence or incidence figures.

When weighing the results in this review, the quality of each individual study is important. However, other characteristics of the study are also of importance. Results from prospective and case-control study designs may offer much more insight in causal mechanisms than cross-sectional designs and should thus receive more attention. In the present review a majority of the studies have prospective designs, following the study population over time. However, many of these studies mainly provide baseline cross-sectional results, due to low incidence of musculoskeletal disorders during follow-up or due to other methodological or practical problems. The time period for data-collection is also of importance, as computer work and computer work stations have had a rapid development. Not all studies report when the data was collected [Additional file 3], which is a drawback as it may take several years from data is collected to a paper is published. One of the included studies was published in 1981 [34] and thus must have data from a very early stage of computerized work. Another study [41] was based on data collected in 1984. The remaining papers were published between 1994 and 2008.

We have chosen to only evaluate the physical exposure in computer work, as we regard psycho-social and organizational exposure factors to be common for many or all kinds of work and not specific for computer work. As a consequence there are several findings in the included studies that are not reported. However, it has been regarded as a plus that such factors have been studied and when appropriate controlled for in the analyses of physical exposure factors.

In this review we have restricted our interest to musculoskeletal disorders that have been diagnosed with a physical examination and not only based on subjective complaints of pain and discomfort. However, it may be argued that some of the diagnoses are in a grey zone between subjective complaints and "objective" clinical diagnoses. This is the case with the diagnosis of clinical forearm case used in the NUDATA study [38], but also with the more common diagnosis of tension neck syndrome. One would suspect that these diagnoses with a relatively high impact of subjective pain report in the diagnostic criteria, would tend to show a relationship to computer work that is more similar to the relationships documented for subjective pain reports.

The following discussion of possible relationships between computer work and musculoskeletal disorders is done for five anatomical regions: neck, shoulders, elbows, forearms and wrists/hands. However, some of the analyses of data in this review are given for even broader regions, such as neck/shoulders, shoulder/arm, etc.

### Neck - tension neck syndrome

Tension neck syndrome, a condition characterized by pain complaints and neck muscle tenderness elicited by palpation and/or movement of the neck, is in this review by far the most common diagnosis in the neck region and is included in the examination protocol of a majority of the included studies. In three studies [26,31,32,40] the diagnosis somatic pain syndrome, with a similar definition, is used. In a prospective study of newly hired computer workers [31,32] hours of keying per week was not associated with incident tension neck syndrome. The baseline cross-sectional analysis in the NUDATA-study [37] showed an increased risk for tension neck syndrome, including an exposure-effect relationship, for work with a computer mouse for more than 15-20 h/w. A similar relationship was not observed for keyboard use. The one-year incidence of tension neck syndrome was too low for reliable analyses even if the NUDATA-study included several thousand subjects. Another much smaller and older study [24,25] found no association between amount of computer work in itself and tension neck syndrome. A community-based case-control study [43] found for women a significant association for shoulder-neck diagnosis (58% of affected subjects had tension neck syndrome) with computer work ≥ 4 hours/day. Several studies of low to moderate quality have found an association between computer work and clinical findings [23,28,30,34]. These studies examine mainly keyboard work. This is supported by a study finding more trigger points and pain provoked by neck sideways flexion in subjects performing data entry work compared with subjects doing data dialogue work [20]. A prospective study of air-traffic controllers changing from varied computer work to a strict mouse-based system [21], only found significant increase of musculoskeletal disorders in the neck and shoulders among the younger half of the study group. At baseline a majority of the affected controllers had tension neck syndrome [52], however there is no information on specific diagnoses at follow-up.

The work-related load of the neck in computer work is influenced by the computer workstation lay-out (including use of specific devices) and individual working technique, and several of the studies in this review have tried to take accord of some of these factors. The NUDATA-study [37] with more than six thousand subjects found no significant associations between tension neck syndrome and several recorded ergonomic factors. Among newly hired computer workers [31,32] a "protective" effect of inner elbow angle above 121° during keyboard use was observed, but this effect was attenuated with increasing hours of keying per week. This study also showed a tendency for increased risk with shoulder flexion above 35° during mouse use, and for a protective effect of the use of chair armrests. In a randomized controlled intervention study [40] a forearm support board was associated with a reduced incidence of neck/shoulder disorders among female call centre operators (tension neck syndrome was found in 59% of the subjects with one or more neck/shoulder diagnoses). However, this relation was not found in a similar randomized intervention study on engineers (male majority) [26], and the NUDATA study [37] gave no support for a protective effect of forearm support on the occurrence of tension neck syndrome. In a study with no observed association to computer work in general, an association to tension neck syndrome was found in subjects with limited rest break opportunities, in subjects who had their keyboard too highly placed relative to elbow level, and in subjects who used bifocal glasses [24,25]. The association of tension neck syndrome to use of bifocals was also shown in another study [33]. Neck flexion more than 20° was identified as a risk factor, however the outcome measure was not precisely described [23].

In a comparison of daily workload by comparing part-time and full-time air-traffic controllers, there was no difference in neck-shoulder or arm-hand disorders [21]. However, a significant effect was observed on subjective complaints from the same body regions, illustrating that an effect seen in complaint scores may not be reflected in the number of diagnoses from a physical examination.

Previous critical reviews that include evidence based on subjective reports of pain and symptoms conclude mostly with a causal relationship between computer work per se (or computer work in general) and neck pain, e.g. [1,5,47,53]. In the NUDATA-study the results on tension neck syndrome were supported by baseline data for neck and shoulder pain symptoms; neck symptoms showed a weaker but still significant exposure-effect relationship to mouse use but not to keyboard use. Some indications were presented that the incident of new neck pain symptoms was associated to mouse use more than 30 h/w and almost significant to keyboard use for more than 15 h/w [37]. Several cross-sectional studies recording subjective pain symptoms only have shown an association between neck and shoulder pain and computer work [54-57]. However, a number of high quality prospective studies do not confirm these findings [58-61]. Aspects of work station design, data equipment and work technique have been shown to influence subjective reporting, such as forearm support for neck symptoms [61-63], and mouse position [54], mouse design [64] and neck flexion angle [65] for neck/shoulder symptoms.

Jensen et al. [66] found a lower number of EMG-gaps and a more repetitive activity on the mouse side compared to opposite side, indicating a more harmful muscle activity pattern on the mouse side. However, increased activity in the trapezius muscle has also been reported after exposure to psychological stress [67-69] and high precision demands [70]. The population at risk is perhaps more prone to a high level of perceived muscular tension [68,71,72], which has been found even when adjusting for high physical exposure, high job strain and age [73]. Several studies document an interaction between mechanical work load in computer work and psychosocial risk factors [58,74].

#### Evidence of a causal relationship for tension neck syndrome?

Of the studies included in this review one cross-sectional study of moderate quality [28] suggests an association between computer work per se and tension neck syndrome. One case-control study of high quality [43] had similar findings, especially for women. One prospective study of high quality [24,25] found no association. With respect to specific aspects of computer work, one very high quality prospective study documents a clear association between mouse use and tension neck syndrome [37]. In a prospective study following a work-task redesign with intensified mouse use, a similar effect was seen in the younger half of the involved workers [21]. In two very high quality intervention trials the introduction of forearm support protected against shoulder-neck diagnoses among female call centre operators [40] but not in among male engineers [26]. Several high quality prospective studies of symptoms do not support an association. Possible pathomechanisms have been documented.

We conclude that there is limited evidence for a causal relationship for computer work per se and for mouse time, but not for keyboard time (Table 1). Several pathophysiological and experimental studies give biological plausibility to this conclusion. However, indications are found of the importance of individual working technique and work station lay-out in causality of tension neck syndrome. These include lack of forearm support, non-neutral position of forearm and neck flexion. This conclusion is in part also a consequence of the limited number of studies.

**Table 1:** Level of evidence for a causal relationship.

Diagnosis	Risk factor		
	**Computer use per se**	**Computer mouse time**	**Computer keyboard time**

Tension neck syndrome	**+**	**+**	**0**
Shoulder tendonitis	**0**	**0**	**0**
Epicondylitis (medial or lateral)	**0**	**0**	**0**
Forearm disorders	**0**	**+**	**0**
Wrist tendonitis	**+**	**+**	**+**

+++ Strong evidence

++ Moderate evidence

+ Limited evidence

0 Insufficient evidence

- Evidence suggesting a lack of causal relation

There is less documentation concerning the relationship between computer work and other neck diagnoses, however, the limited data seem to fit into the pattern illustrated above for tension neck syndrome and thus contribute to the evidence given regarding a possible relationship between computer work and diagnoses in the neck region [22,24,25,40].

### Shoulders - shoulder tendonitis

Many studies put neck and shoulder disorders in one group in the analyses, as mentioned above in the discussion of the neck disorders, making it difficult with a conclusion on shoulder disorders in specific, especially since the neck diagnoses usually were by far the more frequent. When specific diagnoses in the shoulder region are stated, some form of tendonitis is the most common type of diagnosis, and will in the following be labelled shoulder tendonitis. In the NUDATA-study [37] they also diagnosed shoulder myalgia. However, the definition of this disorder overlaps extensively with tension neck syndrome, reducing its specificity as a shoulder disorder. They found no exposure-response relationship or otherwise increased risk for right shoulder myalgia of keyboard or mouse use.

Shoulder tendonitis was one of four in the "shoulder diagnosis group" in the Bergqvist study [24,25,75] and presumably the most common. Data entry operators showed no increased risk for shoulder diagnoses in that study, and not for working hours above 20 h/w, neither for data entry nor interactive operators [24]. Limited rest break opportunity was a risk factor for shoulder diagnoses for all computer workers [25]. In a cross-sectional study supraspinous tendonitis and bicipital tendonitis was only observed among keyboard users and not among the controls [28]. In a cross-sectional study of more than 500 telecommunication workers 29 cases of rotator cuff tendonitis were observed, however, there was no relation to estimated keystrokes per day [33]. The evidence from the two high quality intervention trials cited above under neck disorders [26,40], could just as well have been cited here, as shoulder tendonitis was as frequent as somatic pain syndrome (tension neck syndrome) in the study showing effect of forearm support [40].

As for neck pain, previous critical reviews mostly conclude with a causal relationship between computer work and shoulder pain [1,5,47,53]. Repetitive movements [15] and fixed keyboard height [76] seems to be risk factors, otherwise the documentation is sparse. An exposure-response relationship has been shown for right shoulder symptoms and mouse use, a tendency also for keyboard use but no effect of arm support [37]. Cross-sectional studies have indicated an increased risk for shoulder pain symptoms after four hours daily mouse use [57], and four hours of keyboard use [77].

#### Evidence of a causal relationship for shoulder tendonitis?

One study of moderate quality [28] found an association between computer work per se and supraspinous tendonitis, and one study of high quality [24] found no association. Several studies had too sparse data. We conclude that there is insufficient evidence for a causal relationship for computer work per se, keyboard and mouse time (Table 1).

### Elbows - epicondylitis

Epicondylitis, lateral or medial are the relevant diagnoses in the elbow region that have been included in several of the studies in this review, often grouped together as one category. In the NUDATA-study no association was found between keyboard or mouse use and clinical diagnoses of epicondylitis [39], however they identified relatively few prevalent cases at baseline and very few incident clinical cases during follow-up among the subjects with elbow pain, making a statistical analysis of a possible relationship difficult. A cross-tabulation of the cases with mouse and keyboard time showed no remarkable patterns. The study by Bergqvist et al. [24,25] showed no significant association between epicondylitis and computer work per se. Similarly these diagnoses were not associated with keyboard operators compared to non-keyboard operators in the study by Ferraz et al. [28]. However, only two cases were found in each exposure group, making the study inconclusive. Lateral epicondylitis was more frequent in extensive computer users in a recent cross-sectional study [22].

In an intervention with an alternative mouse design for computer workers with work-related upper extremity pain, the number of subjects with epicondylitis dropped from 2/3 to zero after half a year [78]. In a work-task redesign of air-traffic control from varied computer work to intensified mouse use, the number of arm-hand diagnoses was low before the change and all diagnoses were epicondylitis [52]. After the change the diagnoses in the arm-hand region increased significantly in both male and female controllers, but the specific diagnoses were not specified [21]. However, in the discussion part of the thesis based on this study [79], the author states that the increase in arm-hand problems was mainly localized to the forearm.

Existing reviews diverge concerning conclusions on the evidence for a causal relationship between computer work and elbow pain/epicondylitis [1,15]. Karlqvist et al. [54] found an increased risk of elbow/forearm/hand symptoms with computer work over 2 h/day. In the NUDATA-study a 25% increased odds ratio for severe elbow pain was found above 5 h/w of mouse use, showing a clear exposure-response relationship, but with no threshold effect. Mouse speed, keyboard use or micropauses were not associated with pain [51]. Keyboard use did not show the same pattern. Arm/wrist support did not reduce the risk for severe elbow pain in mouse use, but some beneficial effect was found in keyboard use [39]. The odds ratio for severe elbow pain was increased for continuous mouse time of 10 h/w, but not for continuous keyboard time.

#### Evidence of a causal relationship for epicondylitis?

None of the included studies found association between computer work characterstics and diagnosed epicondylitis, however, only one study [24,25] had conclusive results. We conclude that there is insufficient evidence for a causal relationship for computer work per se, keyboard and mouse time (Table 1).

### Forearms

In the NUDATA-study [38] the odds ratio of being a forearm pain case was eightfold higher if the subject worked more than 30 h/w with a mouse device. Too few new clinical forearm cases during follow-up made it impractical to make analyses on the incident cases.

Karlqvist et al. [80] showed that computer assisted design operators had a 2-4 times greater risk for arm symptoms when using computer mouse for >5.6 h/w compared to less than 5.6 h/w. Operators working with "non-optimal" mouse position reported more symptoms from many regions in the upper extremity. An "optimal" position of the mouse resulted in the lowest muscle activity in the neck, shoulder and arm muscles [81].

#### Evidence of a causal relationship for forearm disorders?

One very high quality study [38] documented an association between the risk for being a forearm pain case and mouse use more than 30 h/w, but this was the only study that investigated this diagnostic entity. There was found insufficient prevalence and incidence rate to conclude for radial nerve compression and pronator teres syndrome [see Additional file 3]. As mentioned above under the discussion of elbow disorders, a significant increase of hand-arm diagnoses in the air-traffic controllers was probably localized to the forearm and related to intense mouse use [79]. We conclude that there is limited evidence for a causal effect of mouse time on forearm pain diagnoses. There is insufficient evidence for a causal relationship for computer work per se and keyboard time (Table 1).

### Wrist/hands - wrist tendonitis

Extensor and flexor tendonopathy/tendonitis and De Quervains syndrome is merged into the diagnostic entity "wrist tendonitis" in this paragraph. A prospective study of newly hired computer workers [31,32] showed a significant 4% increase in risk (hazard ratio) for hand-arm diagnoses for every hour of keying performed per week. A majority of these diagnoses fall in the category of wrist tendonitis, and a third of the cases received their diagnosis within the first month of employment. In this study they also found that a horizontal location of the "J" key more than 12 cm from the edge of the desk was associated with a lower risk of hand/arm disorders (and symptoms). This may be another way of describing forearm support. An elevated position of the keyboard ("J" key more than 3.5 cm above table surface) and a radial deviation for more than 5° while using a mouse were risk factors for hand/arm disorders. Another interesting finding of this study was a doubled risk of hand/arm disorders when using a keyboard wrist rest [32]. In the NUDATA-study with several thousand subjects the number of cases of wrist tendonitis was low both at baseline and at one year follow-up and showed no remarkable pattern with relation to the computer work exposure variables. However, an increased odds ratio for severe wrist/hand pain was found above 5 h/w of mouse use, showing a clear exposure-response relationship, but with no threshold effect [51]. In a community based study examining nearly 1200 subjects with upper extremity disorders, the use of a keyboard or typewriter more than one hour per day increased the risk of wrist tendonitis [45]. As the data was collected in 1998-2000 one might assume that this factor mainly reflects keyboard use. In a cross-sectional study the prevalence of tendovaginitis/tendonitis in the wrist/hand was higher among keyboard users compared to controls [28], as was similarly found for De Quervains syndrome in a recent study [22]. However, no relation to estimated keystrokes per day was observed in a cross-sectional study of telecommunication workers [33]. An exposure-response relationship between risk for arm/hand diagnoses and lowering of the keyboard in relation to elbow level has been observed [24]. The paper does not give information on the distribution of diagnoses in this category. Alternative mouse and forearm support interventions both were associated with reduced incidence of left, but not right, arm-hand disorders [40]. This paper gives the raw frequencies of specific disorders, but as wrist tendonitis probably is less than half of the cases (the number of subjects falling into this broader category is not obvious from the raw data) it is not easy to use the data in our setting. An ulnar deviation (abduction) of the wrist for more than 20° increases risk of clinical findings in the forearm, wrist or hand [34].

The critical reviews that have focused on computer work all concluded with a causal relationship between computer work per se and upper extremity complaints and disorders [1,2,47], however, reviews on generic factors did not support this conclusion [15,82]. Several cross-sectional studies have shown an association between computer work and wrist/hand pain [56,57,77,83]. This is also supported in prospective studies of computer use [58,60] or typing [84].

Forearm support seems also to reduce ulnar deviation in keyboard use [63]. Karlqvist et al. [80] showed an increased risk for arm symptoms when using computer mouse for >5.6 h/w. The introduction of a mouse design reducing hand pronation had beneficial effect on wrist/hand pain in an intervention study [78]. Decreased muscle activity has been found in the hand extensors when working in a neutral hand position [85,86].

A repetitive ulnar deviation task with 20-25 repetitions per minute performed during a working day showed low-frequency fatigue without noticeable discomfort [87]. This has also been found after 10 minutes of static wrist extensions at 10% of maximal voluntary contraction, and with a continued effect after 150 minutes of recovery [88]. Time pressure and verbal provocation during computer mouse use resulted in increased heart rate, blood pressure and muscle activity in neck, forearm and hand muscles [89].

### Evidence of a causal relationship for wrist tendonitis?

One very high quality study [39,51] showed a positive trend between mouse time and risk for wrist extensor tendonitis, and another very high quality study [31,32] showed an exposure-effect relationship for keying time. A community based study of moderate quality showed a relation for daily use of keyboard for more than an hour [45]. One study of high quality [24,25] showed no association with computer work per se, but this study was inconclusive for this specific diagnostic entity [Additional file 3]. We conclude that there is limited evidence for a causal relationship for computer work per se, mouse and keyboard time (Table 1). Several pathophysiological and experimental studies give biological plausibility to this conclusion. Indications exist of a reduced risk for wrist tendonitis with forearm support, a low keyboard and vertical mouse design. An increased risk may be caused by wrist support during keyboarding and ulnar deviation of the wrist. The conclusion is in part also a consequence of the limited number of studies.

Carpal tunnel syndrome (CTS) is not included in the present review, as this diagnostic entity has been studied separately in a parallel review [11]. That review concluded that there is insufficient evidence for a causal relationship for computer work per se, keyboard and mouse time on the development of CTS.

### Further research

Most people in modern working life use computers to a large and increasing extent. Many report musculoskeletal pain, but since the prevalence of work related musculoskeletal diagnoses are low, we need to develop more efficient study designs that may unravel questions concerning causality. One approach would be to conduct case-control studies that include work-related factors (with computer work as one of many relevant factors), as this design is efficient with rare events [90]. Only two of the 22 studies in the present review had this design. More research on epidemiological associations is needed, as well as studies on mechanisms and clinical aspects that focus on a possible effect of computer work on the musculoskeletal system. This includes the possible multifactorial causality of these disorders.

## Conclusions

The main results are summarized in Table 1, showing limited evidence for an association between computer work and some of the studied musculoskeletal disorders. We emphasize that these conclusions are based on few included studies of computer work and diagnostic entities. None of the evidence was considered moderate or strong and there is a need for more and better documentation. The report does not assess the possibility of a causal relationship between this kind of exposure and pain symptoms.

## Competing interests

The authors declare that they have no competing interests.

## Authors' contributions

All authors conceived of the study, took active part in all aspects of the study and read and approved the final manuscript. KBV was the lead author of the original report. MW was the main contributor in the process of up-dating and revising the contents of the original report and in preparing this manuscript.

## Pre-publication history

The pre-publication history for this paper can be accessed here:

http://www.biomedcentral.com/1471-2474/11/79/prepub

## Supplementary Material

Additional file 1 **Table S2. Schematic assessment of the methodological quality of the included studies**. The Table gives the items of the quality assessment list and the scoring of the individual studies included in the review. Click here for file

Additional file 2 **Table S3. Summary of case definition criteria in the included epidemiological studies**. Table gives the criteria for classification of musculoskeletal disorders used in the individual studies. The descriptions of these criteria make reference to six papers [91-96] not cited elsewhere in this review. Click here for file

Additional file 3 **Table S4. Summary of included epidemiological studies**. The Table provides information on study design, population, response rate, physical examination, exposure measures, confounder control, and results relevant for this review presented in the individual studies. Click here for file
