# Supplementary material for: Computer work and musculoskeletal disorders of the neck and upper extremity: A systematic review
Source: BMC Musculoskelet Disord. 2010 Apr 29;11:79. doi: 10.1186/1471-2474-11-79 (PMC2874766; doi:10.1186/1471-2474-11-79)
Supplement: Additional file 1 — Table S2. Schematic assessment of the methodological quality of the included studies. The Table gives the items of the quality assessment list and the scoring of the individual studies included in the review. [file 1471-2474-11-79-S1.DOC]

Table 2. Schematic assessment of the methodological quality of the included studies.

| **Design (a)** | | | **Quality assessment item list** | **Studies:** | Aarås | **Arvidsson** | **Aydeniz** | **Baker** | **Bergqvist** | **Conlon** | **Dainoff** | **Ferraz** | **Ferreira** | **Fogg** | **Gerr/Marcus** | **Hales** | **Hünting** | **Jepsen** | **Konarska** | **NUDATA (f)** | **Rempel** | **Ryan** | **Toomingas** | **Tornqvist** | **Turhan** | **Walker-Bone** |
| --- | --- | --- | --- | --- | --- | --- | --- | --- | --- | --- | --- | --- | --- | --- | --- | --- | --- | --- | --- | --- | --- | --- | --- | --- | --- | --- |
| **Pr** | **Cr** | **Ca** | **Study purpose:** | |  |  |  |  |  |  |  |  |  |  |  |  |  |  |  |  |  |  |  |  |  |  |
| √ | √ | √ | 1 Positive if a specific, clearly stated purpose was described | | 1 | 1 | 1 | 1 | 1 | 1 | 1 | 1 | 0 | 1 | 1 | 1 | 0 | 1 | 1 | 1 | 1 | 1 | 1 | 1 | 1 | 1 |
|  |  |  | **Study population:** | |  |  |  |  |  |  |  |  |  |  |  |  |  |  |  |  |  |  |  |  |  |  |
| √ | √ | √ | 2 Positive if the main feature (description of sampling frame, distribution by age and gender) of the study population  were stated | | 1 | 1 | 1 | 1 | 1 | 1 | 1 | 1 | 1 | 1 | 1 | 1 | 1 | 1 | 1 | 1 | 1 | 0 | 1 | 1 | 1 | 1 |
| √ | √ | √ | 3 Positive if the participation rate at the beginning of the study was at least 80% | | 1 | 1 | 0 | 1 | 1 | 0 | 1 | 1 | 1 | 0 | 0 | 1 | 0 | 1 | 1 | 0 | 0 | 1 | 1 | 1 | 0 | 0 |
| √ |  |  | 4 Positive if the response after 1-year of follow-up was at least 80% or if the nonresponse was not selective | | 1 | 1 |  |  | 1 | 1 | 1 |  | 0 |  | 1 |  |  |  | 0 | 1 | 1 |  | 0 |  |  |  |
|  |  | √ | 5 Positive if the cases and referents were drawn from the same population and a clear definition of the cases and  referents was stated, and if people with chronic upper limb pain (>90 days) are excluded from the controls | |  |  |  | 1 |  |  |  |  |  |  |  |  |  |  |  |  |  |  |  | 1 |  |  |
|  |  |  | **Exposure measurements:** | |  |  |  |  |  |  |  |  |  |  |  |  |  |  |  |  |  |  |  |  |  |  |
| √ | √ | √ | 6 Positive if data on physical load at work were collected and used in the analysis. | | 1 | 1 | 1 | 1 | 1 | 1 | 1 | 1 | 1 | 0 | 1 | 1 | 1 | 0 | 1 | 1 | 1 | 1 | 0 | 1 | 1 | 1 |
| √ | √ | √ | 7 Positive if data on physical load at work were collected using standardized methods of acceptable quality (b) | | 1 | 1 | 0 | 1 | 0 | 0 | 1 | 0 | 0 | 0 | 1 | 0 | 1 | 0 | 0 | 1 | 0 | 0 | 0 | 1 | 0 | 0 |
| √ | √ | √ | 8 Positive if data on the psychosocial factors at work were collected and used in the analysis (c) | | 1 | 1 | 0 | 0 | 1 | 1 | 1 | 0 | 0 | 0 | 1 | 1 | 0 | 0 | 0 | 1 | 1 | 1 | 0 | 1 | 1 | 1 |
| √ | √ | √ | 9 Positive if data on psychosocial factors at work were collected using standardized methods of acceptable quality (b) | | 0 | 1 | 0 | 0 | 0 | 1 | 0 | 0 | 0 | 0 | 1 | 1 | 0 | 0 | 0 | 1 | 1 | 0 | 0 | 1 | 0 | 1 |
| √ | √ | √ | 10 Positive if data on physical and psychosocial load during leisure time were collected and used in the analysis | | 1 | 1 | 0 | 0 | 0 | 1 | 0 | 1 | 0 | 0 | 1 | 0 | 0 | 0 | 0 | 1 | 1 | 0 | 0 | 0 | 0 | 0 |
| √ | √ | √ | 11 Positive if data on historical exposure at work were collected and used in the analysis | | 0 | 0 | 0 | 0 | 1 | 1 | 0 | 1 | 1 | 0 | 1 | 0 | 0 | 1 | 0 | 0 | 1 | 1 | 0 | 0 | 1 | 0 |
| √ | √ | √ | 12 Positive if data on history of upper limb disorders, age and gender were collected and used in the analysis | | 0 | 0 | 0 | 0 | 1 | 1 | 0 | 0 | 0 | 0 | 1 | 0 | 0 | 0 | 0 | 0 | 1 | 0 | 0 | 0 | 0 | 0 |
|  | √ | √ | 13 Positive if exposure assessment was blinded with respect to disease status (d) | |  |  | 1 | 1 |  |  |  | 1 |  | 0 |  | 0 | 0 | 0 |  |  |  | 0 |  | 0 | 0 | 0 |
|  |  | √ | 14 Positive if exposure was measured in an identical way among the cases and referents | |  |  |  | 1 |  |  |  |  |  |  |  |  |  |  |  |  |  |  |  | 1 |  |  |
|  |  | √ | 15 Positive if exposure was assessed at a time prior to the occurrence of the outcome | |  |  |  | 0 |  |  |  |  |  |  |  |  |  |  |  |  |  |  |  | 0 |  |  |
|  |  |  | **Outcome measurement:** | |  |  |  |  |  |  |  |  |  |  |  |  |  |  |  |  |  |  |  |  |  |  |
| √ | √ | √ | 16 Positive if data on outcome were collected using standardized methods of acceptable quality (b) (e) | | 0 | 1 | 1 | 0 | 1 | 1 | 0 | 1 | 0 | 0 | 1 | 1 | 0 | 1 | 0 | 1 | 1 | 0 | 1 | 1 | 0 | 1 |
|  |  | √ | 17 Positive if incident cases were used (prospective enrolment) | |  |  |  | 0 |  |  |  |  |  |  |  |  |  |  |  |  |  |  |  | 0 |  |  |
| √ |  |  | 18 Positive if data on outcome were collected for at least 1 year | | 1 | 1 |  |  | 1 | 1 | 1 |  | 1 |  | 1 |  |  |  | 1 | 1 | 1 |  | 0 |  |  |  |
| √ |  |  | 19 Positive if data on outcome were collected at least every 3 months | | 0 | 0 |  |  | 0 | 1 | 0 |  | 1 |  | 1 |  |  |  | 0 | 0 | 1 |  | 1 |  |  |  |
|  |  |  | **Analysis and data presentation:** | |  |  |  |  |  |  |  |  |  |  |  |  |  |  |  |  |  |  |  |  |  |  |
| √ | √ | √ | 20 Positive if the statistical model used were appropriate for the outcome studied and the measurement of the  association estimated with this model were presented (including confidence intervals) | | 1 | 1 | 1 | 1 | 1 | 1 | 1 | 0 | 1 | 1 | 1 | 1 | 0 | 1 | 0 | 1 | 1 | 1 | 1 | 1 | 1 | 1 |
| √ | √ | √ | 21 Positive if the study controlled for confounding | | 1 | 1 | 0 | 1 | 1 | 1 | 0 | 0 | 1 | 0 | 1 | 1 | 0 | 0 | 0 | 1 | 1 | 0 | 1 | 1 | 0 | 1 |
| √ | √ | √ | 22 Positive if the number of cases in the multivariate analysis was at least 10 times the number of independent  variables in the analysis | | 0 | 1 | 0 | 0 | 1 | 1 | 0 | 0 | 0 | 0 | 1 | 1 | 0 | 0 | 0 | 1 | 1 | 0 | 1 | 1 | 0 | 1 |
| √ | √ | √ | 23 Positive if the study discusses the findings in relation with relevant clinical diagnostic criteria | | 1 | 1 | 1 | 0 | 0 | 1 | 1 | 0 | 1 | 0 | 1 | 1 | 0 | 1 | 0 | 1 | 1 | 0 | 1 | 1 | 1 | 1 |
| 18 | 16 | 20 | **Number of positive items /**  **total number of items** | | 12/ 18 | 15/ 18 | 7/ 16 | 10/ 20 | 13/ 18 | 16/ 18 | 10/ 18 | 8/ 16 | 9/ 18 | 3/ 16 | 17/ 18 | 11/ 16 | 3/ 16 | 7/ 16 | 5/ 18 | 14/ 18 | 16/ 18 | 6/ 16 | 9/ 18 | 14/ 20 | 7/ 16 | 10/ 16 |

(a) This column shows whether the item was used in the quality list for prospective (Pr), cross-sectional (Cr) or case control (Ca) studies. Intervention studies are included in the category prospective studies.

(b) This item was scored positive if the quality of the methods used was tested and documented by the authors or the authors used (and made reference to) well established and documented methods in the literature.

(c) Data on working hours and on pauses from work were assessed as physiological exposure.

(d) If more than one exposure were assessed in the study, it was sufficient that one exposure assessment was blinded to have a positive score on this item.

(e) This item was scored for the data on outcome of the clinical examination.

(f) The score for the NUDATA-study concerns three papers with the first authors Brandt, Kryger and Lassen.
