# Supplementary material for: Computer work and musculoskeletal disorders of the neck and upper extremity: A systematic review
Source: BMC Musculoskelet Disord. 2010 Apr 29;11:79. doi: 10.1186/1471-2474-11-79 (PMC2874766; doi:10.1186/1471-2474-11-79)
Supplement: Additional file 2 — Table S3. Summary of case definition criteria in the included epidemiological studies. Table gives the criteria for classification of musculoskeletal disorders used in the individual studies. The descriptions of these criteria make reference to six papers [91-96] not cited elsewhere in this review. [file 1471-2474-11-79-S2.DOC]

# Table 3. Summary of case definition criteria in the included epidemiological studies.

The table is based on the description given in each study of the criteria for classification of different musculoskeletal disorders.

Studies are listed in alphabetical order after first author, except for the three papers in the NUDATA-study (listed under ‘N’).

| **Ref.** | **Clinical criteria (signs or diagnosis)** |
| --- | --- |
| Aarås et al. (2005) [20] | Used the standardized protocol for the clinical examination in the MEPS collaboration as described in a separate paper [91].  The examination consisted of a general observation of the musculoskeletal system, measurements of the range of passive movements of the neck and head, palpation of muscle spasm and sore spots (trigger points) of the trapezius muscle and measurements of the pressure of the most painful trigger point (value taken when the subject reported serious or radiating pain). Palpation of tendon attachments to supraspinatus and deltoideus for tenderness or pain (performed both with relaxed muscles and against active resistance during muscle contraction). Isometric and endurance tests were performed by lifting the shoulder with arms hanging relaxed and by abduction of the upper arms to 90°. The protocol did not establish musculoskeletal diagnoses (with an exception for carpal tunnel syndrome). |
| Arvidsson et al. (2008) [21] | Clinical criteria as described in Ohlsson et al. (1994) [92], with some additions and modifications that is not in detail outlined in the paper. The following list is provided by the papers’ first author and thus is updated on the points that differ from the original examination protocol of Ohlsson et al.  - Tension neck syndrome: Neck pain; sense of fatigue or stiffness in the neck; pain radiating from the neck to the back of the head; tightness of muscles (in at least 2 out of 4 possible); tender spots in the muscles (in at least 3 out of 8 possible localisations).  - Cervical syndrome: Pain radiating from the neck to the upper extremity; limited neck movement; radiating pain provoked by test movements; decreased sensibility in hands/fingers; muscle weakness of the upper limb.  - Cervicalgia: Neck pain; limited mobility (in at least 4 out of 6 possible directions). Diagnosis only if tension neck syndrome or cervical syndrome is not present.  - Trapezius myalgia: Neck pain; tightness of the muscle; palpable tender spots in the muscle. Diagnosis only if tension neck syndrome or cervical syndrome is not present.  - Thoracic outlet syndrome: Pain radiating of the upper extremity, in the distribution of the ulnar nerve; paresthesia in the distribution of the ulnar nerve; positive Roos’ test (increase of the subjective symptoms, not only fatigue); intense tenderness over the brachial plexus. Diagnosis only if tension neck syndrome or cervical syndrome is not present.  - Frozen shoulder: Shoulder pain; progressive stiffness of the shoulder during the last 3-4 months; limited outward rotation and abduction.  - Supraspinatus tendinitis: Shoulder pain; local tenderness over the tendon insertion; pain at resisted isometric abduction.  - Infraspinatus tendinitis: Shoulder pain; local tenderness over the tendon insertion; pain at resisted isometric outward rotation.  - Bicipital tendinitis: Shoulder pain; local tenderness over the tendon(s); pain at resisted isometric elevation of the arm (straight and elevated 90º) and/or resisted isometric flexion of the elbow (flexed 90º and hand supinated).  - Acromioclavicular syndrome: Shoulder (epaulet) pain; palpable tenderness of the joint; pain provoked by horizontal adduction and/or by outward rotation of the arm (90º abducted, with flexed elbow).  - Lateral/medial epicondylitis: Elbow pain; palpable tenderness of the lateral and/or medial epicondyle; pain at resisted isometric extension or flexion of the wrist; for the diagnosis lateral epicondylitis, pain and/or weakness in gripping.  - De Quervain’s disease: Wrist pain; palpable tenderness of the tendons of the first dorsal compartment of the wrist; positive Finkelstein’s test.  - Peritendinitis/tenosynovitis: Wrist pain; palpable tenderness of the tendon(s); local swelling; redness or heat.  - Overused hand syndrome: Hand pain; palpable tenderness of the thenar, hypothenar and interossei muscles, and of the wrist capsule.  - Carpal tunnel syndrome: Nocturnal numbness of the hand; paresthesia in the distribution of the median nerve; positive Tinel’s sign over the carpal tunnel and/or positive Phalen’s test and/or decreased sensibility in the distribution of median nerve and/or decreased strength in opposition of the thumb.  - Pronator teres syndrome: Pain of the medial/proximal part of the forearm; local tenderness over the edge of m. pronator teres; pain and decreased in pronation and/or decreased flexion strength of the wrist and/or of the distal phalanxes of the fingers I-II.  - Frohse’s syndrome (radial tunnel syndrome): Pain in the elbow during rest; pain of the proximal, lateral part of the forearm; tenderness about 5-8 cm distally of the lateral epicondyle and pain at resisted isometric supination.  - Ulnar nerve entrapment (at the elbow): Pain and paresthesia or numbness in the distribution of the ulnar nerve; positive Tinel’s sign over the cubital tunnel.  - Ulnar nerve entrapment at the wrist: Pain and paresthesia or numbness in the distribution of the ulnar nerve; decreased sensibility of the fingers IV-V and/or positive Tinel’s sign over Guyon’s tunnel (volar/ulnar at the wrist) and/or decreased strength in spreading the fingers. |
| Aydeniz and Gursoy (2008) [22] | Clinical examination by a physician from the department of physical medicine and rehabilitation, following a standardized protocol for the neck and upper extremities: (The authors’ descriptions of the clinical tests are included in parentheses.)  - Cervical degenerative diseases: Neck range of motion test and Spurling’s test (extending and rotating the neck and applying downward pressure on the hand).  - Biciptal tendinitis: Yergason’s test (positive if pain in the bicipital groove with the elbow flexed 90° and forearm pronated against resistance) and Speed’s test (positive if pain in the bicipital groove in resistive shoulder flexion with the subjects elbow extended and forearm supinated).  - Lateral epicondylitis: An epicondyle stretch test (resistive extension of the wrist).  - Cubital tunnel syndrome: Cubital tunnel test (positive if paresthesia and pain provoked by sustained elbow flexion combined with gentle digital pressure on the cubital tunnel).  - De Quervain’s tenosynovitis: Flinkelstein’s test (stabilizing the forearm and instructing the subject to make a fist with the thumb tucked inside the other fingers – pain proximal to the thumb on ulnar deviation indicates de Quervain’s tenosynovitis).  - Carpal tunnel syndrome (CTS): Tinel’s sign (positive if tingling pain in the distribution of the median nerve upon percussion over the palmar aspects of the wrist) and Phalen’s test (maintaining maximal voluntary wrist flexion for 1 min). Positive results of the Tinel’s sign and Phalen’s tests indicated CTS.  References for the provocation tests are given in the paper. |
| Baker  et al. (2008)  [23] | The cases had to have been diagnosed by a physician with any musculoskeletal disorder in the upper extremities believed to be associated with computer use. The physical examination was not a part of the study. The examination was thus not standardized and performed by several different physicians. |
| Bergqvist  et al.  (1995)  [24,25] | Clinical physiotherapeutic examination as described in Wolgast (1989) [75] with specific criteria for the following diagnoses, requiring three or more symptoms or signs. (Pains due to diabetes, inflammatory joint diseases or traumata were excluded).  - Tension neck syndrome: Ache/pain in the neck, tiredness and stiffness in the neck, possible headache, pain during movements, muscular tenderness.  - Cervical syndrome: Ache/pain in the neck and arm, headache, decreased mobility due to cervical pain during isometric contraction, often root symptoms such as paresthesia or numbness.  - Cervical degenerative disease: Ache/pain in the neck, possibly headache, decreased mobility due to pain during movements.  - Frozen shoulder: Ache/pain in the shoulder (especially at night time), decreased mobility, pain during movements.  - Shoulder tendonitis: Ache/pain in the shoulder, decreased mobility, pain from isometric contraction of the muscle involved.  - Degenerative disease of the glenohumeral joint: Ache/pain in the shoulder, decreased mobility, pain at passive movements, palpation tenderness.  - Degenerative disease of the acromoclavicular joint: Ache/pain in the shoulder, pain during arm movements above shoulder height, palpation tenderness.  - Medial epicondylitis: Ache/pain in the elbow, palapation tenderness of the medial epicondyle, pain during resisted flexion of the hand.  - Lateral epicondylitis: Ache/pain in the elbow, palapation tenderness of the lateral epicondyle, pain during resisted extension of the hand.  - Carpal tunnel syndrome: Ache/pain in the hand, paresthesia in the part of the hand innervated by the median nerve, positive Phalen’s test. - Tenosynovitis/tendonitis in the hand/wrist: Ache/pain in the hand, activity induced pain, resisted muscle test pain, swelling, muscle weakness and painful tendon palpation.- Degenerative joint disease in the hand: Joint ache/pain, sensation of tiredness and stiffness in the hand, decreased mobility, swelling. |
| Conlon et al. (2008) [26] | Used the examination protocol of Gerr et al. (2002), see below |
| Dainoff et al. (2005) [27] | Used the standardized protocol for the clinical examination in the MEPS collaboration, see Aarås et al. (2005) above. |
| Ferraz et al. (1995) [28] | Musculoskeletal and neurological examination by a rheumatologist with criteria for the following diagnoses:  - Tension neck syndrome: Feeling of fatigue and/or stiffness in the neck, headache or neck pain, at least 2 tender spots or palpable hardenings, or muscle tightness in neck movements.  - Bicipital tendonitis: Localized shoulder pain with tenderness over the bicipital tendon.  - Supraspinous tendonitis: Localized shoulder pain with tenderness over the supraspinous tendon.  - Epicondylitis: Localized elbow pain with tenderness on the lateral or medial epicondyle.  - Wrist tendosynovitis/tendonitis: Localized tenderness and/or swelling of the tendon and sheat of the flexor carpi radialis or the extensor carpi.  - Carpal tunnel syndrome: History of pain, paresthesias, and/or numbness in the fingers or palm, positive Phalen’s or Tinel’s sign, and/or observation of atrophy or weakness. The diagnosis had to be confirmed by electromyography on median-nerve testing.  - Myalgia: Pain in the muscles in the absence of physical findings suggestive of inflammation.  - Arthralgia: Pain in the joints in the absence of physical findings suggestive of inflammation. |
| Ferreira et al. (1997) [29] | Hands and/or wrists diagnoses were based on pain history and/or physical examination of tendon or tendon sheath or from nerve entrapment. All cases had been examined by an occupational physician and a physician trained in rheumatology or orthopaedics. Criteria for specific diagnoses are not given in the paper, neither are figures for the number of cases in different diagnostic entities. |
| Fogg and Henderson  (1996) [30] | No defined diagnoses were presented in the article. The physician made an assessment of the severity of the pain on a five-point Likert type rating scale and decided whether the subject displayed no strain, postural strain or repetitive strain. |
| Gerr et al. (2002) and Marcus et al. (2002)  [31,32] | Standard physical examination of symptomatic body region (and corresponding contralateral body region) by an occupational therapist, with the following case definitions:  - Radicular pain syndrome: Positive neck compression test (i.e., Spurling`s test).  - Somatic pain syndrome: Pain on palpation of either sternomasteoid muscle or trapezius muscle and abnormal cervical range of motion (criteria specified in the paper).  - Rotator cuff tendonitis: Supraspinatus point tenderness (compared with contralateral side and had to be greater on symptomatic side) AND either positive supraspinatus muscle test or painful arc motion test.  - Bicipital tendonitis: Point tenderness on palpation of the long head of the biceps AND either positive Speed`s test or positive Yergason`s test  - Lateral epicondylitis: Positive Cozen`s test OR positive Mill`s manoeuvre AND positive lateral epicondyle point tenderness or positive lateral (extensor) muscle mass tenderness.  - Medial epicondylitis: Positive Reverse Cozen`s test AND either a positive medial epicondyle point tenderness or positive medial (flexor) muscle mass point tenderness.  -Flexor carpi radialis tendonitis: Pain at the volar radial side of the wrist with resisted radial deviation and wrist flexion AND one of following symptoms: point tenderness, local warmth, swelling, redness or crepitance.  - Flexor carpi ulnaris tendonitis: Pain at the volar ulnar side of the wrist with resisted ulnar deviation and wrist flexion AND one or more of the symptoms: point tenderness, local warmth, swelling, redness or crepitance.  - Digital flexor tendonitis: Pain at the palmar wrist with resisted wrist and digit flexion AND one or more of the symptoms: point tenderness, local warmth, swelling, redness or crepitance.  - Extensor tendonitis – dorsal compartment 1 (abductor pollicis longus & extensor pollicis brevis): Positive Finkelstein’s test or pain on resisted thumb metacarpal phalangeal extension.  - Extensor tendonitis – dorsal compartment 2 (extensor carpi radialis longus & extensor carpi radialis brevis): Pain on the dorsum of the hand at the base of second and third metacarpal with resisted wrist extension and radial deviation AND one or more of the following findings: point tenderness, local swelling, warmth, redness, or crepitance.  - Intersection syndrome: Two of the following three findings: point tenderness located on the dorsolateral side of the wrist proximal to the extensor retinaculum, localized swelling proximal to the extensor retinaculum, and crepitance.  - Extensor tendonitis – dorsal compartment 3 (extensor pollicis longus): Pain on resisted thumb IP extension AND one or more of the following findings: point tenderness, local swelling, warmth, redness, or crepitance.  - Extensor tendonitis – dorsal compartment 4 (extensor digitorum communis (EDC) & extensor indicis propriis (EIP)): With interphalangeal joints flexed, the subject extend and flex the metacarpal phalangeal joint 5-10 times. Pain on the dorsal aspect of the wrist or distal forearm with resisted digit extension with the interphalangeal joints flexed (EDC) or on the dorsal radial side of the wrist with isolated sesisted index finger extension (EIP) AND one or more of the following findings: point tenderness, local swelling, warmth, redness, or crepitance.  - Extensor tendonitis – dorsal compartment 5 (extensor digiti minimi): Pain on the dorsal ulnar side of the wrist with isolated resisted small finger extension AND one or more of the following findings: point tenderness, local swelling, warmth, redness, or crepitance.  - Extensor tendonitis – dorsal compartment 6 (extensor carpi ulnaris): Pain on the dorsal ulnar side of the wrist with resisted ulnar deviation and wrist extension AND one or more of the following findings: point tenderness, local swelling, warmth, redness, or crepitance.  - Distal flexor tenosynovitis (trigger finger): Pain in the flexor tendon sheath at the A1 pully and either crepitance in the flexor tendon sheath at the A1 pully or decreased range of motion of digit due to locking in either flexion or extension.  - Carpal tunnel syndrome: Paresthesias in the distribution of the median nerve and prolonged sensory latency (3.2ms at 14cm distance) of the median nerve across the affected wrist.  -Ulnar neuritis: Paresthesias in the distribution of the ulnar nerve and prolonged sensory latency (3.2ms at 14cm distance) of the ulnar nerve.  References for the provocation tests are given in the paper. |
| Hales et al. (1994) [33] | Physical examination by specially trained physicians for the following diagnoses:  - Tension neck syndrome: Pain on resisted flexion, extension, or rotation. Palpation of spasms or trigger points in the trapezius muscle.  - Cervical root syndrome: Positive Spurling’s test.  - Rotator cuff tendinitis: Pain at active resisted arm abduction ≥90°, painful deltoid palpation  - Bicipital tendinitis: Positive Yergason`s test.  - Thoracic outlet syndrome: Positive hyperabduction and Adson`s test.  - Epicondylitis: Pain at palpation of medial or lateral epicondyle.  - Proximal tendinitis: Pain in proximal 2/3 of the forearm when resisted wrist or finger flexion or extension.  - Radial tunnel syndrome: Positive Mill’s test.  - Distal tendinitis: Pain in distal 2/3 of the forearm or in the hand on resisted wrist or finger flexion or extension.  - De Quervain`s disease: Positive Finkelstein`s test.  - Carpal tunnel syndrome: Positive Tinel’s and Phalen’s tests.  - Guyon tunnel syndrome: Positive Tinel’s test (over the Guyon tunnel).  - Ganglion cysts: Presence of ganglion cysts.  - Joint-related: Decreased MCP or PIP range of motion (<100°)  - Trigger finger: Locking of finger in flexion or palpable tendon sheath ganglion  References for the provocation tests are given in the paper. |
| Hünting et al. (1981) [34] | The medical examination included an anamnesis and palpation of painful pressure points in shoulders, arms and hands. Furthermore, painful symptoms in isometric contractions of the forearm were assessed. |
| Jepsen and Thomsen (2006)  [35] | A physical examination on selected neurological parameters following a protocol developed and validated by the lead author in previous publications [93-95] was performed by an examiner blinded to any patient-related information. The examination assessed manually the isometric motor function in 11 contractions, performed in three standard positions and testing the function of the pectoral, posterior deltoid, latissimus dorsi, biceps, infraspinatus, and triceps muscles, and the radial flexor of wrist, abductor of small finger, short abductor of thumb, short radial extensor of wrist and ulnar extensor of wrist. Algesia for needle prick was assessed in five and threshold to vibration in three innervations territories. Mechanosensitivity (soreness) of nerve trunks was assessed at seven locations by palpating with moderate manual pressure. Three characteristic patterns of physical findings were defined in accordance with the topography of the upper limb nerves and their muscular and cutaneous innervation. The employed criteria for each location were the following:  - Brachial plexus neuropathy, chord level: Weakness during the contractions reflecting the deltoid, biceps, and radial flexor of wrist muscles accompanied by increased threshold for vibration in the median nerve-territory, and soreness with a moderate pressure over the infraclavicular brachial plexus at chord level.  - Posterior interosseous neuropathy: Weakness during the contraction reflecting the ulnar extensor of wrist muscle and soreness with a moderate pressure over the posterior interosseous nerve at the arcade of Frohse.  - Median neuropathy, elbow level: Weakness during the contraction reflecting the radial flexor of wrist muscle and increased threshold of vibration in the median nerve-territory, and soreness with a moderate pressure over the median nerve at elbow level. |
| Konarska et al. (2005) [36] | Used the standardized protocol for the clinical examination in the MEPS collaboration, see Aarås et al. (2005) above. |
| NUDATA-study  Brandt et al. (2004) [37]  Kryger et al.  (2003) [38]  Lassen et al.  (2004) [39] | Clinical examination of symptom cases by a physician using a standardized interview and examination protocol for the following diagnoses. (Subjects with surgery or trauma, or with a medical condition that could explain the symptoms were not included.)  - Tension neck syndrome: Pain and stiffness in the neck with palpation tenderness in the trapezius muscle.  - Rotator cuff syndrome: Pain in the deltoid region of the upper arm on interview, a positive impingement test, pain on resisted abduction, external rotation or internal rotation.  - Shoulder myalgia: Substantial palpation tenderness in the levator scapula, the supraspinous or the infraspinous muscles.  - Clinical forearm case: Moderate/severe palpation tenderness in proximal aspect of the forearm  - Supinator syndrome: Substantial pressure palpation tenderness over the fibrous arch at the origin of the supinator muscle, pain in the same area with resisted supination of forearm, and/or resisted extension of the middle finger.  - Pronator teres syndrome: Substantial pressure palpation tenderness on the volar side of the proximal forearm, pain in the pronator area with resisted pronation of the forearm, and/or paresthesias in dig 1-3 with resisted flexion of the middle finger.  Reference for the provocation tests is given in the paper.  - Lateral epicondylitis: Pain at the lateral epicondyle or the neighbouring soft tissue, palpation tenderness over the epicondyle, pain with resisted extension of the wrist. The pain caused at least “quite a lot of trouble” during the last year.  - Medial epicondylitis: Pain at the medial epicondyle or the neighbouring soft tissue, palpation tenderness over the epicondyle, pain with resisted flexion of the wrist. The pain caused at least “quite a lot of trouble” during the last year.  - Wrist tendonopathy: Hand/wrist pain located to the extensor or flexor tendons combined with either tendon point tenderness or swelling or crepitation.  - De Quervain’s syndrome: Radial wrist pain, point tenderness located to the first dorsal compartment and pain in the same area with passive ulnar deviation of the wrist with the thumb fixed (Finkelstein’s test – reference is given in the paper). |
| Rempel et al. (2006) [40] | Used the examination protocol of Gerr et al. (2002), see above. |
| Ryan and Bampton (1988)  [41] | The paper gives no spesific information and only states that a physical examination of the neck, shoulders and arms was carried out, seeking areas of muscle tenderness or hardening. In the results sections the authors state that they had not found any cases of tenosynovitis or epicondylitis (medial or lateral) and that the most appropriate diagnosis for their findings was myalgia (diffuse muscle pain and tenderness). |
| Toomingas et al. (2003)  [42] | The paper gives very little specific information on the medical examination and no reference for the examination protocol used by the physicians. The examination of the musculoskeletal system covered inspection, active and passive range of movement, pain at resisted movement, and palpation tenderness. Pathological findings were classified into about 140 different predefined conditions (separating left and right side when possible). |
| Tornqvist et al. (2000) [43] | A standardized and reliability tested examination protocol was used and the following diagnoses are stated in the article, but no definition is given:  - Tension neck syndrome  - Cervical brachialgia  - Shoulder tendonitis |
| Turhan  et al. (2008)  [44] | The paper gives no specific information on or reference for the examination protocol used. All subjects were examined by the same physician who was blinded to the background of the subjects. The examination consisted of a test battery including tests to diagnose ganglions, De Quervain’s disease, extensor tenosynovitis, medial and lateral epicondylitis, bicipital tendonitis and thoracic outlet syndrome. |
| Walker-Bone et al. (2006) [45] | Subjects with pain were examined according to the standardized Southampton examination schedule [96] and classified into one of the following diagnoses or were classified as having non-specific pain:  - Rotator duff tendonitis: History of pain in the deltoid region AND pain on resisted active movement (abduction – supraspinatus,; external rotation – infraspinatus; internal rotation – subscapularis)  - Adhesive capsulitis: History of pain in the deltoid area AND equal restriction of active and passive gleohumeral movement with capsular pattern (external rotation > abduction > internal rotation).  - Bicipital tendonitis: History of anterior shoulder pain AND pain on resisted active flexion or supination of forearm.  - Subacromial bursitis  - Acromioclavicular joint dysfunction  - Lateral epicondylitis: Epicondylar pain AND epicondylar tenderness AND pain on resisted extension of the wrist.  - Medial epicondylitis: Epicondylar pain AND epicondylar tenderness AND pain on resisted flexion of the wrist.  - Tenosynovitis at the wrist: Pain on movement localised to the tendon sheaths in the wrist AND reproduction of pain by resisted active movement.  - De Quervain’s disease: Pain over the radial styloid AND tender swelling of first extensor compartment AND EITHER pain reproduced by resisted thumb extension OR positive Finkelstein’s test.  - Osteoarthritis of thumb base or distal interphalangeal joints |
