# Supplementary material for: Computer work and musculoskeletal disorders of the neck and upper extremity: A systematic review
Source: BMC Musculoskelet Disord. 2010 Apr 29;11:79. doi: 10.1186/1471-2474-11-79 (PMC2874766; doi:10.1186/1471-2474-11-79)
Supplement: Additional file 3 — Table S4. Summary of included epidemiological studies. The Table provides information on study design, population, response rate, physical examination, exposure measures, confounder control, and results relevant for this review presented in the individual studies. [file 1471-2474-11-79-S3.DOC]

# Table 4. Summary of included epidemiological studies.

The papers are listed in alphabetical order after first author, except for the three papers in the NUDATA-study (listed under ‘N’). Confidence limits are 95%.

| **Study Country / year of data-collection** | **Design, population, response/ participation rate (R)** | Control group | **Physical examination (Clinical criteria –**  **see Table 3 [Additional file 2])** | **Exposure measures/indicators relevant for this review (physical factors)** | **Confounder control** | **Results relevant for this review** |
| --- | --- | --- | --- | --- | --- | --- |
| Aarås et al. (2005) [20]  Norway  Year of data-collection not stated | One year follow-up study of 90 computer workers (data entry: 30 females; data dialog: 30 males and 30 females).  R follow-up 58% (52/90). | No control group. | Physical examination performed before and after intervention and after one year follow-up. | Intervention with optometric corrections, ergonomic information and training, and limited small-scale ergonomic adjustments. Workload assessed before and after intervention by EMG, inclinometry and observation. | Personal characteristics, social conditions and psychosocial work characteristics. | The clinical examination revealed no significant changes within the groups during the study period. The data entry group had significantly more findings in the examination at baseline, compared to the other two groups. |
| Arvidsson et al. (2008) [21]  Sweden  Baseline: 2002-2003  Follow-up: 2006-2007 | Five year follow-up study of 187 air-traffic controllers (38% females).  R baseline 100%,  R follow-up 79% (148/187). | No control group. | Physical examination by the same physiotherapist performed at baseline and follow-up. | A change in air-traffic control system from a system implying varied computer work to a strictly mouse-based system was implemented 20 months before follow-up, with physical workload of neck/upper extremity (EMG, inclinometry) measured in both systems in a subgroup of 7 male and 7 female controllers. | Questionnaire on psychosocial work conditions and interview on leisure time exposure collected both at baseline and follow-up. | Prevalence of elbow/hand diagnoses 3.4% → 10% (p=0.02), same tendency in men (2.6% → 9.1%) and women (4.3% → 11%).  Prevalence of shoulder/elbow/ hand diagnoses higher in right vs left arm at follow-up (16% vs 9.5%, p=0.04), but not at baseline (8.8% vs 6.8%, p=0.61). |
| Aydeniz and Gursoy (2008) [22]  Turkey  Year of data-collection not stated | Cross-sectional study of 100 office workers (41% females) with >6h computer work/day for >2 years.  Number invited to the study not stated. | 65 office workers (48% females) with <2h computer work/day. | Physical examination by physician blinded to exposure status. | Group level: >6h computer work/day for >2 years contrasted to <2h computer work/day.  Individual level: Cumulative computer use time (no information on how it was measured). | Age and gender. | Prevalence in study group versus control group:  Cervical degenerative disease: right 21% vs 9%, left 8% vs 3%.  Biciptal tendonitis: right 8% vs 3%, left 11% vs 5%.  Lateral epicondylitis: right 8% vs 3%, left 11% vs 5%.  Cubital tunnel syndrome: right 13% vs 2%, left 3% vs 0%.  De Quervain’s tenosynovitis: right 22% vs 2%, left 5% vs 0%.  All differences significant with exception of left cubital tunnel syndrome (p=0.079). |
| Baker  et al. (2008)  [23]  USA  Year of data-collection not stated | Case-control study with all subjects recruited from university faculty and staff. 21 cases (86% females) with physician-diagnosed computer-associated upper extremity disorder.  Number invited to the study not stated. | 21 controls matched to the cases for age (±5 years) and gender. | Physical examination not part of the study (diagnosis from their own physician inclusion criteria for cases) . | Personal computer keyboarding style scored with a 19-item instrument (covering static posture, dynamic posture and force) in one minute video clip (left, right and overhead views) when the subjects typed a standard text on their own computer workstation. | Age and gender. | In a classification and regression tree (CART) analysis a model with neck flexion angel ≥ 20° separated cases from controls with a low sensitivity (0.48), but high specificity (0.90), accuracy = 69%. In a separate analysis for neck disorders only (6 cases, 21 controls) neck flexion angel ≥ 20° separated cases from controls with a perfect sensitivity (1.00), and high specificity (0.90), accuracy = 93%. |
| Bergqvist  et al. (1995)  [24,25]  Sweden  Baseline: 1981  Follow-up: 1987 | Six year follow-up study of 353 office workers remaining at the workplace (34% (182/535) dropout during follow-up).  R follow-up 92% (questionnaire) and 91% (physical examination). | The material consisted of 52% interactive, 29% data entry and 19% non computer users. | Physical examination by physiotherapist performed at follow-up only. | Observed ergonomic factors (9 items). Self-reported computer use. | Personal characteristics (including age, gender, BMI), psychosocial factors. | Keyboard too highly placed was associated with tension neck syndrome (OR=4.4 (1.1-17.6)).  Limited rest breaks opportunities associated with tension neck syndrome (OR=7.4 (3.1-17.4)) and shoulder diagnoses (OR=3.3 (1.4-7.9)).  Arm/hand diagnoses were associated to computer work ≥ 20 h/week when combined with limited rest opportunity and non-use of lower arm support (OR=4.6 (1.2-17.9)) and to a combination of non-use of lower arm support and limited opportunities for rest breaks (OR=10.1 (2.4-43.2)). |
| Conlon et al. (2008) [26]  USA  2002-2003 | One year follow-up of 206 engineers (28% females) randomised to three intervention groups and one control group.  R inclusion 47% (206/437),  R follow-up 45% (92/206). | (see previous column) | Physical examination by physician blinded to exposure status. Only subjects meeting symptom criteria on a weekly survey had an examination, starting from week 4 after intervention. | The subjects were randomised to receive (1) a conventional mouse, (2) an alternative mouse with a neutral forearm posture, (3) the conventional mouse plus a forearm support board, or (4) the alternative mouse plus the forearm support board. Weekly survey on work schedule and hours of computer use. | Personal characteristics, work history, psycho­social work conditions, physical/mental health history, and leisure time activities. | No statistically significant effects of the interventions on incident musculoskeletal disorders (one year incidence of neck/shoulder/ arm disorders of 20.3%). |
| Dainoff et al. (2005) [27]  USA  Year of data-collection not stated | One year follow-up of 29 female data entry workers.  R follow-up: 90% (26/29). | No control group. | Physical examination by physician before and after intervention. The physician performing post-intervention examinations was not aware of previous findings. | Intervention consisting of optometric corrections, ergonomic information and training, and ergonomic adjustments | Organizational and psychosocial factors at work, leisure time psychosocial factors and physical activity. | Change from pre test till one year follow-up:  Reduced number of painful trapezius triggerpoints (Friedman=198.69, p<0.001)  Improved scores on shoulder function tests (χ^2^(2df)=817.5, p<0.001)  Improved scores on neck mobility tests (χ^2^(2df)=429.4, p<0.001) |
| Ferraz et al. (1995) [28]  Brazil  Year of data-collection not stated | Cross-sectional study of  130 keyboard operators (54% females).  Participation rate:  97% (130/134). | 133 traditional office workers (41% females) .  Participation rate:  84% (138/165). | All subjects received a brief physical examination by physiotherapist. Subjects with signs or reporting symptoms received a full evaluation by a rheumatologist blinded to exposure status. | Length of employment, self-reported break time, and mean keystroke performance preceding month for each subject. | Personal characteristics, work history, stress at work, ergonomic factors, satisfaction with the workstation, amount of leisure time physical activity, general health. | Keyboard users had significantly more hand/wrist tendonitis (p<0.001), tension neck syndrome (p<0.01) and supraspinatos tendonitis (p<0.02).  All diagnosis grouped together were associated to length of employment and < 30min break time. No association with keystroke performance.  (Carpal tunnel syndrome was the diagnosis in 2/50 cases in keyboard user group and in 0/12 cases in the control group.) |
| Ferreira et al. (1997) [29]  Brazil  1993-1995 | Dynamic cohort of female call centre operators studied retrospectively for 2½ years. Population at risk was adjusted monthly (mean n=46, min n=30, max n=72, total number of subjects in the study 106). | No control group. | All cases had been examined by an occupational physician and a specialist in rheumatology or orthopaedics | Interventions on rest-work schedule, work-organization and ergonomic outline during the 2½ year study period. | Age, seniority, overtime, regular work time. | The implementation of 10 min/hour rest break reduced the risk of musculoskeletal disorders in the hand and wrist.  (Figures for specific diagnoses not reported, including carpal tunnel syndrome.) |
| Fogg and Henderson  (1996) [30]  New Zealand  Year of data-collection not stated | Cross-sectional study of 512 keyboard operators.  Number invited to the study not stated. | 561 non-keyboard clerical workers. | Subjects reporting pain were offered a physical examination by a physician. | Keyboard vs non-keyboard users from the same offices. | Unclear if/how the results were adjusted for personal characteristics and health data. | Keyboard users displayed more repetitive strain injuries (χ^2^(1df)=42.20, p<0.05) |
| Gerr et al. (2002) [31]  Marcus et al.  (2002) [32]  USA  1995-1998 | Up to 3 year follow-up of 632 newly hired computer workers (71% females).  R 66% (632/956). | No control group. | Symptom cases identified at baseline or during follow-up were offered a physical examination by an occupational therapist. | Characteristics of workstation layout and work posture:  9 keyboard use items  5 mouse use items  6 other workstation items  Diary self-reported daily hours of computer use | Personal characteristics, psychosocial factors, smoking, health history, previous computer use. Occupational stress measured 4 weeks after enrolment. | Neck/shoulder disorders:  No association with hours keying per week (HR per hour =1.01 (0.99-1.03)).  Reduced risk when working with an inner elbow angle >121° (HR=0.11 (0.02-0.66)). This effect diminished with increasing keying hours.  Arm/hand disorders:  Associated with hours keying per week (HR per hour =1.04 (1.02-1.06)).  Reduced risk with keyboard “J” key >12 cm from the tables edge (HR=0.38 (0.20-0.71)).  Increased risk using keyboard wrist rest (HR=1.96 (1.05-3.65)).  Increased risk with >5° of wrist radial deviation in mouse use (HR=1.82 (1.03-3.22)). |
| Hales et al. (1994) [33]  USA  Year of data-collection not stated | Cross-sectional study of  533 telecommunication workers having >6h/day computer work.  R 93% (533/573). | No control group. | Physical examination by a physician blinded to case status was offered all subjects | The subjects belonged to 5 distinct job categories. Automatically monitored keystroke information was available for a subset of 174 workers. | Work organization and psychosocial factors, leisure time activities, general health and demographic data. | Keystrokes/day was not associated to musculoskeletal disorders in any of the four anatomical regions (neck, shoulder, elbow, and hand/wrist).  Only hand/wrist disorders showed an association with job category. |
| Hünting et al. (1981) [34]  Switzerland  Year of data-collection not stated | Cross-sectional study of  162 computer workers: 53 data entry (94% females) and 109 data dialog (50% females).  Number invited to the study not stated. | 78 fulltime typist (95% females) and 55 office workers with little use of keyboard (60% females). | Physical examination by a physician | Body posture and workplace dimensions | Unclear if/how the results were adjusted for age, gender and psychosocial factors. | In computer users: Wrist ulnar abuction >20° associated with increased risk.  Data entry workers and fulltime typists had increased risk for isometric contraction pain. |
| Jepsen and Thomsen (2006)  [35]  Denmark  Year of data-collection not stated | Cross-sectional study of 96 computer workers (engineers and technical assistants, 41% females) using computer > 20% of working hours.  R 82% (96/117). | Mouse operating limb and non-mouse operating limb was compared within subjects. | Physical examination by examiner blinded to patient status. | Mouse operating limb vs non-mouse operating limb. | Within subject analysis. | Relation between mouse / non-mouse operating limbs fulfilling the criteria for:  - brachial plexus neuropathy at cord level (9/2)  - posterior interosseous neuropathy (14/8)  - median neuropathy at elbow level (13/5) |
| Konarska et al. (2005) [36]  Poland  Year of data-collection not stated | One year follow-up of 33 female data entry workers.  R follow-up: 48% (16/33). | No control group. | Physical examination by physician before and after intervention. | Interventions consisting of optometric corrections, ergonomic information and training, and ergonomic adjustments. | Organizational and psychosocial factors at work, leisure time psychosocial factors and physical activity. | No significant change in clinical findings. |
| NUDATA-study  Brandt et al.  (2004) [37]  Kryger et al.  (2003) [38]  Lassen et al.  (2004) [39]  Denmark  2000-2001 | One year follow-up of 6943 technical assistants and machine technicians (63% females) identified through union files.  R questionnaire baseline 73% (6943/ 9480) and follow-up 82% (5658/6943).  R clinical examination baseline:  neck 82% (530/645), shoulder: 85% (395/467), forearm 85% (235/275),  elbow and hand/wrist 82% (1366/1666).  R clinical examination follow-up :  neck 74% (46/62), shoulder 80% (63/79), forearm 82% (49/60), elbow and hand/wrist 75% (327/436). | No control group. | Symptom cases at baseline or incident symptom cases at follow-up were invited to an examination by a physician. | Self-report h/week of computer work (5 categories), active mouse use and active keyboard use. Self-report ergonomic workplace factors (including keyboard/mouse position, forearm support). | Personal characteristics, physical / psychosocial workplace factors. | At baseline tension neck syndrome and weekly mouse use showed a dose-response relationship with RR=2.1 (0.7-6.1), 2.8 (0.9-8.4), 3.5 (1.0-12) and 4.7 (1.2-18), when weekly mouse use increased from 15-19, 20-24, 25-29 to >30 hours.  At baseline using computer mouse ≥30 hours pr. week gave an OR of 8.2 (1.5-43.5) for being a clinical forearm case. |
| Rempel et al. (2006) [40]  USA  2001-2003 | One year follow-up of 182 call centre operators (95% females) randomised to three intervention groups and one control group.  R inclusion: 68% (182/269),  R follow-up 69% (125/182). | (see previous column) | Physical examination by physician blinded to exposure status. Only subjects meeting the symptom criteria had an examination. | The subjects were randomised to receive (1) a conventional mouse, (2) an alternative trackball mouse, (3) the conventional mouse plus a forearm support board, or (4) the alternative mouse plus the forearm support board. Weekly survey on work schedule and hours of computer use. | Baseline questionnaire on demographic data, work history, psycho­social work conditions, physical/mental health history, smoking, and leisure time activities. | Neck/shoulder disorders - forearm support intervention associated with reduced incidence (adj. model: HR=0.49, CI 0.24-0.97).  Left arm/hand disorders - trackball intervention associated with reduced incidence (adj. model: HR=0.19, CI 0.04-0.90) and a tendency for the forearm support intervention (adj. model: HR=0.29, CI 0.08-1.05).  Right arm/hand disorders - no effects observed. |
| Ryan and Bampton (1988)  [41]  Australia  1984 | Cross-sectional study of 143 data process operators (proportion of males and females not stated)  R 99%. | No control group. | Physical examination of all subjects. | Ergonomic assessment of angles and distances for each operator’s working postures. | Psychosocial and physical work conditions, general health. | The 41 operators with the highest combined upper limb symptoms score were compared the 28 with lowest score. Of 10 ergonomic measures there were significant differences in 3:  Left elbow angle: 93°(12°) vs 85°(10°), t:2.84, p:0.006  Left forward arm flexion: 12°(9°) vs 7°(7°), t:2.27, p:0.027  Eye copy distance in mm: 601(78) vs 550(80), t:2.59, p:0.012 |
| Toomingas  et al. (2003)  [42]  Sweden  Year of data-collection not stated | Ten monthly follow-ups of 70 call centre operators (50% females).  R baseline:  f: 83% (29/35), m: 80% (28/35).  Average R at follow-up:  f: 68% (197/290), m: 71% (199/280).  R clinical examination:  f: 100% (5/5), m: 69% (9/13).  R controls baseline:  f: 88% (756/860), m: 79% (470/599),  at follow-up:  f: 88% (6635/7560), m: 86% (4047/4700),  at clinical examination:  f: 76% (239/315), males 75% (150/199). | 1459 computer workers (not at call centres). The controls were older, had more education and more experience with computer work than the call centre operators | Incident symptom cases (had to free of symptom reporting in the preceding questionnaire) were invited to a physical examination by a physician. | No exposure data reported. | Age, education, seniority and experience with computer work. | No significant findings. |
| Tornqvist  et al. (2000)  [43]  Sweden  1994-1997 | Community based case-control study with 392 cases (70% females) and 1511 controls (56% females). Cases were all subjects from the study base who sought care or treatment for neck or shoulder disorders by any caregiver in the region.  R controls: 69%. | The controls were selected at random in the study base (all subjects having residence and being gainfully employed in the municipality of Norrtälje). | All subjects had a standardized physical examination. | Computer work >4h/day | Personal characteristics, physical and psychosocial work and leisure time exposure factors. | Females: Neck and shoulder disorders RR 1.9 (1.0-3.4)  Males: No relation to computer work observed. |
| Turhan  et al. (2008)  [44]  Turkey  Year of data-collection not stated | Cross-sectional study of 173 data entry operators (92% females).  Number invited to the study not stated. | No control group. | All subjects had a physical examination by physician blinded to background data. | All subjects were observed for awkward working postures. | Age, gender, working years, BMI, co-morbid conditions and perceived stress. | The following risk factors for specific disorders were observed:  Leaning wrists on keyboard - extensor tenosynovitis (OR 3.12 (1.05-3.70)).  Excessive thumb extension - De Quervain’s disease (OR 2.72 (1.41-5.26)).  Hard keystrokes - ganglion (OR 3.12 (1.39-7.00)).  Typing with ulnar deviation - ganglion OR 3.12 ((1.41-6.90)). |
| Walker-Bone et al. (2006) [45]  England  1998-2000 | Community based cross-sectional study of 10,264 adults (age 25-64 years) registered in two general practices.  R questionnaire 59% (6038/10264),  R physical examination 62% (1197/1922). | No control group. | Subjects reporting pain were offered a physical examination by a research nurse or physiotherapist blinded to exposure status. | Use of keyboard or typewriter for <1, 1-4, >4 h/day (questionnaire) | Personal characteristics, physical and psychosocial work and leisure time exposure factors. | Typing > 1 h/day increased the risk of tenosynovititis at the wrist (OR 3.2, CI 1.3-7.8). |
